# A prime-masked ERP investigation on phonology in visual word processing among bilingual speakers of alphasyllabic and alphabetic orthographies

**DOI:** 10.1038/s41598-022-13654-8

**Published:** 2022-06-14

**Authors:** Adhvika Shetty, Sanjana P. Hebbar, Rajath Shenoy, Varghese Peter, Gopee Krishnan

**Affiliations:** 1 Manipal College of Health Professions, Manipal Academy of Higher Education, Manipal, Karnataka 576104 India; 2 School of Health and Behavioural Sciences, University of the Sunshine Coast, Sippy Downs, Australia

**Keywords:** Cognitive neuroscience, Human behaviour

## Abstract

In this study, we experimentally manipulated the phonology of the cross-script prime-target dyads in an ERP-coupled masked priming paradigm to explore the role phonology plays in visual word processing. The written characters of certain bilingual dyads seldom show any visual/orthographic similarity, yet have the same phonological representation. While the Bilingual Interactive Activation (BIA) model relies on the orthographic similarity between the languages in a bilingual dyad, its revised version (BIA + model) additionally banks on the phonological (and semantic) similarity between the words in such dyads. Thus, there exists the need to investigate the role of phonological (and semantic) similarity between the words of a bilingual dyad, especially in the absence of orthographic similarity. Borrowed words from one language to another provide a suitable avenue to explore this question. Cross-orthographic (or cross-script) bilingual participants of this study performed the semantic judgment of visually presented words in a masked priming paradigm in each of their languages while we simultaneously collected the event-related potentials (ERPs). The primes were either translations (different phonology & orthography: P–O–; phonologically incongruent) or transliterations (same phonology & different orthography: P + O–; phonologically congruent) of the target. Overall, the results showed no difference between the two prime conditions. We discuss our findings in light of the BIA and BIA + models of bilingual visual word processing and discuss the relevance of the former model in orthographically distinct bilingual language dyads.

## Introduction

A strong body of research proposes that bilinguals activate their non-target language while processing the target language^1–5^. Several studies emphasize the role of phonology in bilingual word processing^1^. However, some highlight the importance of the script in bilingual visual word processing, especially in orthographically distinct bilingual dyads^6,7^. In the current study, we experimentally manipulated the phonology of the prime-target dyads in a bilingual masked priming paradigm (clubbed with event-related potentials) where the two languages do not share any visual or orthographic similarity. We discuss our findings in light of the Bilingual Interactive Activation (BIA^8,9^ & BIA + ^10^) models (see below) that propose the commencement of visual word processing from the letter features.

The Bilingual Interactive Activation (BIA) model^8,9^, and its revised version (BIA +)^10^ are two influential models that explain the visual word processing in bilinguals. These models propose an integrated bilingual lexicon in which words in a language are processed in a non-selective manner^10^. The BIA models follow a bottom-up processing pattern commencing from the letter features to letters with their unique positions within the words and thus to the specific words. The activated words at the word level feed-forward activations to the language nodes, which in turn, would serve as *language tags*, and these tags inhibit the activated words in the non-target language in the previous (word) level through inhibitory connections. The language nodes, however, do not have any mechanism to facilitate language-specific lexical access at the early stages of visual word processing^11^.

The BIA models primarily rely on the orthographic similarity between the languages of bilingual speakers. In the revised version (BIA +) of the model, Dijkstra and van Heuven^10^ implemented phonological (sound level) and semantic (meaning) similarity and proposed that in *addition* to the orthographic similarity, phonological and semantic similarities between the languages also contribute to the bilingual visual word processing. The phonological and semantic similarities between the words are primarily mediated by the orthographic overlap. Several studies^12–14^ have shown such influence at various levels (orthographic overlap, phonological overlap, and semantic overlap), thus supporting the BIA and BIA + models.

While the BIA and BIA + models propose an orthography-driven visual word processing in bilinguals, where the language nodes are activated much later in the process, how does the visual word processing take place in the case of cross-script bilingual contexts, especially when the two scripts do not have any visual resemblance?

A recent study by Peleg et al.^15^ reported on phonological mediation in cross-script (Arabic-Hebrew) bilinguals. Native Arabic speakers, relatively proficient in Hebrew, were able to reject Spoken Arabic (SA) words (that do not have scripts; hence written in Hebrew and served as non-words in that language), in a Hebrew lexical decision task (LDT) compared to non-words in both languages. The authors concluded that such an early rejection of SA words in a Hebrew LDT was due to the sub-lexical phonological mediation from Hebrew to SA. In light of the BIA models, it remains unclear how the Hebrew letters and their positions activated SA words, especially when the latter did not have the script. Further, it may be noted that the participants in Peleg et al.^15^ study had ample time to derive the whole word phonology (and perhaps, the *semantics*) of the non-words (i.e., SA words) from Hebrew letters. Methodologically, to alleviate the derivation of whole-word phonology or any such strategic processing, the masked priming paradigm is recommended^16^.

In a masked priming paradigm, the primes are presented subliminally in a flanked manner (often) by a series of ‘#’ or a random string of letters^16^ to restrict their conscious processing^17^. Even such masked and briefly presented primes influence the targets in visual word processing. In cross-lingual masked priming experiments, primes that are manipulated at various levels (e.g., orthographic^12^, phonological^18^, and translations^19^) all have shown to influence the target processing. Though most of these studies have been conducted in same-script bilinguals, even studies in cross-script bilinguals have shown such influence in visual word processing^20^. An inherent limitation of the masked priming paradigm, however, is that the behavioral outcome measures (e.gs., response time & accuracy) are obtained only at the end of each trial, and thus are not truly reflective of the experimentally controlled (e.g., orthographic or phonological) processes alone. In other words, these outcome measures would also include certain task-dependent processing costs such as the decision-making process and manual response time^12,21^. To circumvent these inherent limitations, investigators have used additional techniques that allow online monitoring of the ongoing processes under investigation. One such technique is the event-related potentials (ERPs).

ERPs have been used in the explorations of visual word processing to monitor the neural dynamics while the participants process the visually presented targets. This technique allows the online monitoring of the brain’s activity at the millisecond level. The three ERP components commonly studied in visual word processing are the N/P150, N250, and N400 responses. The early ERP response P150 (or N150 depending on the electrode location) is understood to indicate low-level visual processing of the stimuli. In support of this notion, N/P150 is modulated by the nature of the stimuli (orthographic vs non-orthographic^22^), font size and type^23^, and letter position^24^. The cognitive process reflected by the N/P150 component is the mapping of visual features onto the pre-lexical orthographic representations^25^. The N250 effect, on the other hand, has been attributed to various pre-lexical processes such as sub-lexical processing of printed letter strings^26^, mapping of prelexical form representations to whole-word form representations, and the orthographic to phonological mapping^27^. This effect has also been reported to arise from an orthographic mismatch between primes and targets^28^. However, the influence of phonological similarity between words, especially in a cross-script bilingual dyad, remains unknown. The N400, a late ERP component has been considered as an index of semantic processing in many masked priming ERP studies^29–31^.

In a cross-script study that clubbed ERPs with non-cognate translation priming, Hoshino and colleagues^29^ reported the N250 effect from Japanese primes (L1) to English targets (L2), but not in the reverse order. While such an effect was not expected in both directions due to the word-form mismatch (i.e., Japanese & English), the authors attributed the observed effect in the L1-to-L2 direction to the feedback from semantic representations activated by the stronger L1 primes to the weaker L2 targets. Hoshino et al. thus maintained that the N250 effect arises from semantic feedback in cross-script non-cognate translation priming contexts.

Chauncey et al.^31^ studied French(L1)-English(L2) bilinguals on a masked priming paradigm where the prime-target pairs were unrelated words that belonged to either the same or different languages. In the ERPs, during the N250 (175-300 ms) and N400 (375-550 ms) time windows, they found an effect of the target language (L1 targets had higher N250 and N400). When the targets were in L2 and the primes were in L1, the N250 was larger and N400 was weaker. The latter finding was interpreted as the code-switching cost. In light of the BIA model, the authors argued that the prime activates the corresponding language node, and this activation sends inhibition to the lexical representations in other language. Since the L1 primes are processed faster than L2 primes, they have an earlier influence on target processing, thereby generating stronger code-switching effects for L2 targets in the N250 time-window.

While the studies reviewed above show that the non-target language’s phonology is active while processing words in the target language^15^ or phonological activation is weaker when primes are in L2^29^, several other studies propose the influential role of orthography in cross-script bilingual dyads. For instance, Gollan et al.^6^ proposed that differences in the script may provide powerful cues to the search in the appropriate lexicon of bilinguals. That is, a prime in Hebrew would provide stronger cues to the subsequently presented targets in Hebrew than in English. ERP evidence to support this claim was recently reported in Spanish–English bilinguals^32^. Hoversten and colleagues^32^ employed an oddball paradigm in which the participants were required to press one button for rare targets in L1 and another button for similar targets in their L2. While the participants performed this task, the ERPs associated with the nonwords (non-targets) that, in turn, appeared like targets were measured. They hypothesized that the ERP indices of infrequent stimuli in an oddball paradigm (e.gs. N2 & P3 components) would be present on the target-like nonwords only if their orthographic cues are processed in the lexical decision task. Both behavioral and ERP findings from this study provided evidence for the early (i.e., pre-lexical) usage of orthographic cues. The target-like non-words (i.e., orthographically similar to the targets) received more false alarms compared to those that did not show such similarity with the targets. As predicted, they observed the N2 and P3 components suggestive of early processing of the orthographic cues. Finally, a very recent study^7^ that compared the cross-script (Japanese-English) and same-script (Spanish–English) bilinguals on a picture-word interference paradigm provided further evidence for the perceptual differences between orthographies as a language cue which can facilitate faster processing of words in the target language.

As stated earlier, according to the BIA models, the visual word processing commences from the early identification of the letter features^8–10^. In light of these models, those cross-script studies that proposed the activation of the phonology of the non-target language while processing words in the target language indicate that the orthography of the former language is activated by the target language. However, other studies maintain that cross-script primes conveniently limit the visual word processing to the relevant (target) language. A primary question per the BIA models, here, is that how do the features of letters in the target language that do not share any visual similarity with letters in the non-target language activate the latter. One way to explore this question is to use the cross-script cognate translation (e.g., borrowed words: henceforth, transliteration) and cross-script non-cognate translation (henceforth, translation) primes during visual word processing. The transliterations are phonologically similar but orthographically dissimilar to each other (P + O–). The borrowed words from one language to another (that do not have a unique translation in the latter language) qualify these conditions. Such words are written with the script of the recipient language, yet spoken akin to the lender language. The translations, on the other hand, are neither phonologically nor orthographically similar (P–O–). That is, such words have a unique word form in each language. Both transliterations and translations share the same semantics (S +) between the languages.

## Current study

In the current study, we administered the masked priming paradigm coupled with ERP on a group of Malayalam-English bilinguals. Malayalam (the primary language spoken in Kerala, a southern state in India) belongs to the alphasyllabic (or semi-syllabic) orthography, like most other Indian languages. Consonantal graphemes in this language are always syllabic, whereas canonical vowel graphemes are phonemic in pronunciation. Further, the visuospatial organization of graphemes in this language is complex compared to English (alphabetic orthography)^33^. More importantly, due to historical reasons, Malayalam, like many languages in India, borrowed several words from English, thus providing us an opportunity to compare the transliteration (i.e., semantically and phonologically similar, but orthographically dissimilar) and translation words as primes to explore the relevance of orthography in cross-orthographically distinct language dyads.

In this study, we hypothesize that if the phonology influenced the visual word processing in cross-script bilingual contexts, the transliteration trials (P +O–, e.g., CAR- {ka:r}﻿) shall yield faster response time (RT) compared to the translation trials (P–O–, e.g., TABLE– {me:ʃa}). This, in turn, would support the BIA + model^10^. On the other hand, a lack of difference in RT between the two conditions would support the BIA model^8,9^, and raise concern over the influence of phonology (and thus, on the BIA  + model^10^) in cross-script bilingual visual word processing contexts. Further, such a lack of difference could augment the argument on the role of orthography in cross-script bilingual visual word processing contexts.

Regarding the ERPs, as the primes and targets are always in different languages, a comparison between the translation and transliteration trials in the early time window (100–200 ms), where the visual/orthographic features are extracted, shall not yield any difference (i.e., no N/P150 effect). More importantly, if phonology influenced the mapping of orthography to phonology in bilingual visual word processing, the N250 effect could be expected (within 200–350 ms) due to phonological mismatch between transliteration trials (P +O–) and translation trials (P–O–). Finally, as both the translation and transliteration trials share the same semantic concepts, we do not expect a difference between translation and transliteration trials in the later time window (i.e., no N400 effect).

## Results

### Behavioral measures

**Figure 1:**
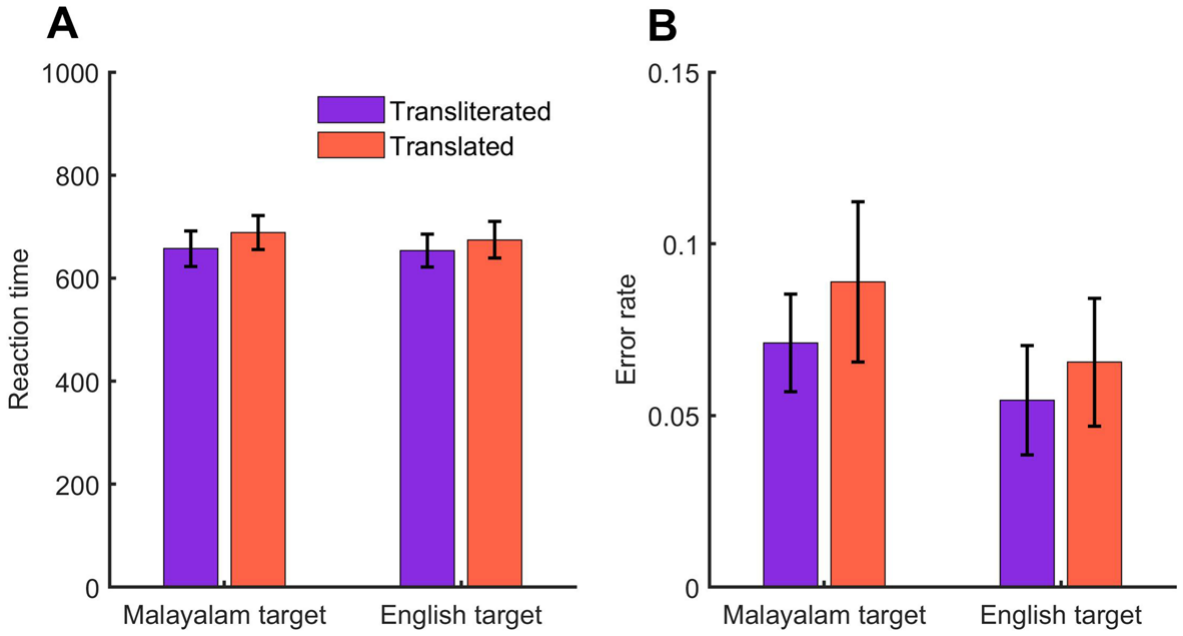
Reaction times (**A**) and error rate (**B**) across the conditions. Error bars represent standard error of mean.

The reaction times and error rates are shown in Fig. 1. We performed separate repeated measures ANOVA on the reaction time and error rates. Though the Transliteration trials elicited faster response time (mean difference = 26 ms) compared to the Translation trials, this difference was not statistically significant (*F*(1, 17) = 3.46, *p* = 0.08, *ŋ*_*p*_^2^ = 0.16). Similarly, we did not observe a significant main effect of target language (*F*(1, 17) = 0.26, *p* = 0.61, *ŋ*_*p*_^2^ = 0.01), though the participants were 9 ms faster while processing English targets compared to Malayalam targets. And, the interaction between prime congruency and target language was not significant (*F*(1, 17) = 0.2, *p* = 0.66, *ŋ*_*p*_^2^ = 0.01).

The results of error data analysis were different from that of the RTs. Participants committed more errors on Translation trials compared to the Transliteration trials (*F*(1, 17) = 5.42, *p* = 0.03, *ŋ*_*p*_^2^ = 0.24). However, neither the main effect of the target language (*p* = 0.16) nor the interaction between prime congruency and the target language (*p* = 0.64) were statistically significant.

### ERP measures

**Figure 2:**
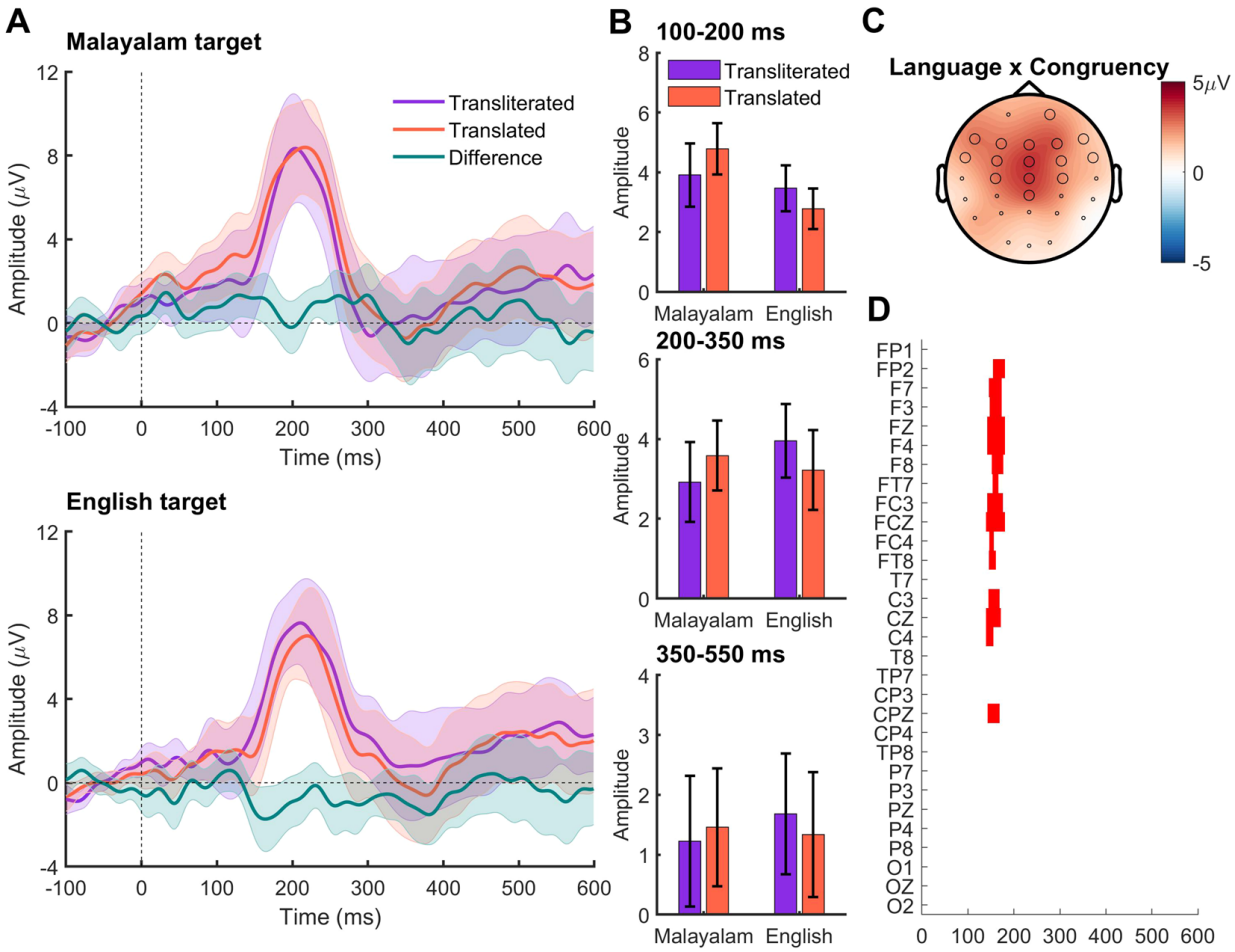
(**A**) ERPs across the conditions. The solid lines represent grand averaged ERPs and the shading encompasses 95% confidence intervals. (**B**) The mean amplitude calculated from 9 electrodes in the central scalp location across different time windows. The error bars represent standard error of mean. (**C**) The topography of the language x congruency effect at 155 ms. The highlighted electrodes belong to a statistically significant cluster (**D**) *p* values masked for significance (*p* < .05) for the language x congruency interaction effect on the basis of the cluster-based permutation test. The x axis represents the time (in milliseconds and y axis represents the 30 recording electrodes).

The grand averaged ERPs for each condition and stimuli are shown in Fig. 2. As expected for visual stimuli, the ERPs showed a positive peak around 200 ms for all stimuli at the central location.

### Literature-driven analyses

#### N/P150 (100 to 200 ms epoch)

The 2 × 2 repeated measures ANOVA computed with the factors Target language (English, Malayalam) and Congruency (Transliteration, Translation) on the amplitude between 100 and 200 ms revealed no significant main effect of the Target language (F(1,17) = 3.80, *p* = 0.068, *ŋ*_*p*_^2^ = 0.18) or prime Congruency (F(1,17) = 0.150, *p* = 0.70, *ŋ*_*p*_^2^ = 0.01). However, the interaction between language and congruency was significant (F(1,17) = 5.59, *p* = 0.03, *ŋ*_*p*_^2^ = 0.25). To further understand the interaction, separate one-way ANOVAs were computed for each language with the factor congruency. These ANOVAs revealed main effect of congruency for the Malayalam targets (F(1,17) = 5.12, *p* = 0.037, *ŋ*_*p*_^2^ = 0.23), where Translation trials generated significantly larger positive response (M = 4.78, SE = 0.86) compared to Transliteration trials (M = 3.90, SE = 1.06). The effect of Congruency for the English targets was not significant (F(1,17) = 2.57, *p* = 0.13, *ŋ*_*p*_^2^ = 0.13). Further, acknowledging that the translations have two phonological word forms (one in each language) unlike the transliterations (that have only one form in the lending language: English here), we computed two separate ANOVAs for each level of Congruency with Language as the factor. These results revealed main effect of Language only in Translation trials (F(1,17) = 14.02, *p* = 0.002, *ŋ*_*p*_^2^ = 0.45), where Malayalam translations again showed significantly larger positive amplitudes (M = 4.78, SE = 0.86) compared to their English counterparts (M = 2.77, SE = 0.67). The mean amplitude of the ERPs in this time window did not differ between Malayalam and English transliterations (F(1,17) = 0.27, *p* = 0.61, *ŋ*_*p*_^2^ = 0.02). Thus, the ERPs showed independent effects of Language and Congruency primarily due to the Malayalam translations in the 100–200 ms epoch.

#### N250 (200 to 350 ms epoch)

The 2 × 2 ANOVA on the amplitude between 200 and 300 ms revealed no significant main effects (Target language: F(1,17) = 0.25, *p* = 0.62, *ŋ*_*p*_^2^ = 0.01; Congruency: F(1,17) = 0.02, *p* = 0.92, *ŋ*_*p*_^2^ = 0.001). However the interaction between Target language and Congruency was significant (F(1,17) = 4.84, *p* = 0.04, *ŋ*_*p*_^2^ = 0.22). But the follow up one-way ANOVA computed for each target language did not show any significant effects for the factor Congruency (Malayalam: F(1,17) = 1.86, *p* = 0.19, *ŋ*_*p*_^2^ = 0.10; English: F(1,17) = 3.23, *p* = 0.09, *ŋ*_*p*_^2^ = 0.16). Here again, like in the previous epoch, we computed two separate one-way ANOVAs for each level of Congruency with Language as the factor. However, both analyses did not show any significant main effects of Language (Transliteration: F(1,17) = 1.61, *p* = 0.22, *ŋ*_*p*_^2^ = 0.08; Translation: F(1,17) = 0.33, *p* = 0.57, *ŋ*_*p*_^2^ = 0.02). Therefore, the ERP amplitude between 200 and 350 ms was similar for the translated and transliterated stimuli in both languages.

#### N400 (350 to 550 ms epoch)

The 2 × 2 repeated measures ANOVA on the ERP amplitudes in the N400 time window (350–550 ms) did not show any significant main effects (Target language: (F(1,17) = 0.44, *p* = 0.83, *ŋ*_*p*_^2^ = 0.003; Congruency: (F(1,17) = 0.12, *p* = 0.92, *ŋ*_*p*_^2^ = 0.001) or interactions (F(1,17) = 0.23, *p* = 0.64, *ŋ*_*p*_^2^ = 0.013) suggesting similar ERP amplitudes across the languages and prime congruencies in this time window.

### Data-driven analysis

The cluster-based ANOVA that included all 30 electrodes and all time points between 0 to 600 ms did not show any significant main effects. However, the interaction between Target language and Congruency was significant between 140 and 180 ms (cluster *p* value = 0.041; Cohen’s d = 0.83). The difference in the ERP between translated and transliterated trials was more positive for the Malayalam targets than for the English targets between 140 and 180 ms (Fig. 2).

## Discussion

In the present study, we compared the behavioral and electrophysiological data from a group of Malayalam-English bilinguals while they processed the prime-target pairs that were either translations or transliterations. The participants performed the semantic judgment of prime-masked target words separately in each language. The two languages considered in this study belonged to entirely different orthographies having minimal or no visual/orthographic similarity between them. As mentioned in the Introduction, while the transliterations share the same phonology and semantics between the languages but not the orthography, the translations share only semantics but not the phonology and orthography. Thus, the primary difference between these two types of words lies in their phonology, and this allowed us to precisely manipulate the phonology between the primes and targets.

The reaction time of the target words preceded by transliterated (i.e., congruent: P+ O–) primes was shorter (i.e., faster) by 26 ms compared to that of the targets preceded by translation (i.e., incongruent: P–O–) primes. However, the difference was not significant. This primarily indicates that the phonological similarity in the case of transliterated primes did not have an influence on the target word processing. This finding is in contrast to the earlier reports on phonological facilitation^15,18^. Note that, in the current study, the orthography of primes always differed from that of the targets. Further, the prime and target had the same semantic representation (i.e., translation & transliteration). While the transliterated primes were phonologically congruent with the target word, the translated primes were phonologically incongruent with the target words. Therefore, the behavioral findings from the current study show that in the absence of orthographic similarity, the phonological similarity between primes and targets did not have any influence on the target word processing. That is, the possible phonological facilitation effect reported elsewhere^34^ could be annulled at subliminal prime display times when the prime’s orthography differs from that of the target. The findings from this study may, therefore, strengthen the arguments on the plausible role of orthography in bilingual visual word processing, at least, in orthographically distinct languages^6^.

The ERP findings of this study mostly endorsed the behavioral data. The only ERP effect observed was between 100 and 200 ms, where (1) the prime congruency had an effect on Malayalam targets, but not on English targets, and (2) the Target language had an effect on Translation (P–O–) trials, but not on Transliteration (P + O–) trials. A closer look at these results reveals that both effects arose from significantly higher ERP amplitude of the Malayalam translations (4.78 µV) over Malayalam transliterations (3.9 µV) and English translations (2.78 µV). These effects were also present in the fully data-driven cluster-based permutation analysis. We did not predict an effect in this time window as the dissimilarities between prime and target were equivalent. What could have caused the rise in the amplitude of Malayalam translation trials? Is this an effect of phonology?

The N/P150 effect observed in the 100–200 ms time window could be attributed to the effect of phonology, as we manipulated this variable in the current study. Though N/P150 effect has been reported in a cross-script bilingual dyads^29^, there are a few caveats in attributing this effect to phonology in the current study, as we outline here. First, we observed this effect only in one direction, that is, when the English primes preceded the Malayalam targets, and not in the reverse order. Second, this effect was observed primarily due to the larger ERP amplitude of the Malayalam translation targets compared to the Malayalam transliteration and English translation targets (see Results: 100–200 ms ERP epoch). If the phonological overlap between the primes and targets were the underlying mechanism, such an effect should have been observed when the Malayalam transliteration primes preceded English targets. However, that was not observed in the current study. It may be noted that the phonological effects in ERPs are reported in the later time windows (250–350 ms & 350–450ms^35^), and the earlier time window shows (e.g., 150–250 ms) the orthographic effects ^25,35,36^. The unidirectional difference in ERP amplitude from English-to-Malayalam translation prime-target dyads indicates that certain additional factor(s) might have influenced the ERP amplitude in this time window. In the following section, we discuss the possible reasons for these observations.

One possible explanation for this effect is that, in the current study, the loaner language was always English. That is, borrowed words were always Malayalam transliterations of English words, but not in the reverse direction (as such words are extremely meager in this bilingual dyad). Thus, the Malayalam translations had a unique lexical form in each language (e.g.,  /me:ʃa/—TABLE). However, this was not the case with Malayalam transliterations as these words had only one word form (in the lending language: here, English) shared with the borrowing language (e.g., CAR- {ka:r}‍). Further, the Malayalam targets were always preceded by English primes. Thus, in the case of translated Malayalam targets, the English primes would have pre-activated their word forms (e.g., TABLE) followed by the word forms in Malayalam (e.g.,  /me:ʃa/—table) by the targets after 500 ms (i.e., duration of the backward mask). These two distinct word forms would have led to certain conflict, giving rise to an increased ERP amplitude (note that there exists ERP evidence for the early access to lexical features (i.e., 100–200 ms) such as word frequency and lexicality while processing visually presented letter strings^37^). On the other hand, in the case of transliteration trials, both prime and target activate the same word form, giving rise to no such conflicts, thus leading to no difference in ERP amplitude. However, this sort of an elevation in ERP amplitude happened only with translated Malayalam targets, but not when they were presented as primes (followed by English unique targets). This indicates that the difference in the word form similarity between the transliteration and translation trials alone cannot explain the observed ERP effect in the 100–200 ms time window. Below, we discuss additional factors that could have possibly influenced the ERP amplitude in this time window.

First, it may be noted that, behaviorally, the Malayalam targets showed a non-significantly longer RT compared to English targets. Second, Malayalam is orthographically (and visually) more complex compared to English^33^. Finally, the participants in this study (applicable to the majority of the youngsters in India who are enrolled in English medium schools), despite their native fluency in speaking and reading Malayalam, had limited exposure to the written form of this language compared to written English early from the kindergarten level. Note that, at the time of their recruitment to this study, the participants were immersed in a fully English dominant (academic) environment for a minimum of 2–6 years, where formal exposure to written Malayalam was absent. The increased error rates with Malayalam targets in our participants (see Results) substantiate this reasoning. Considering all these points above, we believe that the briefly presented English primes might have been processed more effectively, compared to Malayalam primes, at subliminal levels, giving rise to the ERP effect in the case of translated English–Malayalam prime-target pairs in this time window. However, this needs confirmation with additional investigations.

The ERPs in the subsequent time windows (i.e., 200–350 ms & 350–550 ms) also failed to reveal any significant effect of either the prime congruency or the target language. That is, the ERPs failed to show both the N250 and N400 effects. The former effect is considered as an index of orthography-to-phonology mapping processing, whereas the latter is believed to represent semantic processing^25^. In the current study, we hypothesized that as both translation and transliteration prime-target dyads share the same semantic representation, a comparison would yield null difference, and thus, an absent N400 effect. The ERPs in the final time window (350–550 ms) supported our prediction. However, more interesting is the ERP findings in the middle time window (200–350 ms), where the N250 effect was expected. Importantly, and in accordance with the behavioral data from this study, the middle time window did not show any significant effect (i.e., an absent N250 effect), thus, failing to show any influence of phonological similarity between the primes and targets in transliterations compared to translations in each language. While the earlier studies^26,27^ attributed the N250 effect to sub-lexical processing of orthographic stimuli, finding from the 200–350 ms time window in current study is indicative of the non-phonological nature of this effect. Further, it may be noted that those studies that showed an N250 effect have either compared semantically congruent and incongruent prime-target pairs^28,29^ or only used semantically incongruent prime-target pairs^31^. Therefore, it may be the case that semantic (and not phonological) congruency needed to be manipulated to observe an N250 effect.

Consolidating the observations, thus far, it becomes apparent that the RT failed to show any influence of phonology, which in turn was corroborated by an absent N250 ERP effect. These findings, in general, did not support the influence of phonology in bilingual visual word processing context. Based on the behavioral and electrophysiological findings from the current study, we argue that in the absence of orthographic overlap between the primes and targets, phonological (or combined phonological + semantic) similarity may not influence the visual word processing, at least, in orthographically distinct bilingual dyads, such in the current study. These findings could augment the arguments that early orthographic cues might guide visual word processing in a resource-economic, language-selective manner^6,7,32^. Finally, the ERP difference in the early time window (100–200 ms) with translated Malayalam targets and the overall increased error rates with Malayalam targets in this study could possibly be due to the difference in participants’ exposure between written forms of Malayalam and English. In light of these observations, we recommend that future studies on visual word processing in bilinguals shall consider the participants’ exposure to the written forms of the languages under consideration.

### Implication on BIA models

The findings from this study provide certain insights on the BIA models. As delineated in the Introduction, the BIA model^8,9^ relied primarily on the orthographic similarities between the languages in a bilingual lexicon. The findings from the current study are explainable based on this model. For instance, the orthographic features of the target language are initially identified. According to the BIA model^8,9^, the orthographic features of the target language inhibit the neighboring, less-activated units of the non-target language and the activation of the former progresses to the word and then to the language nodes. These language nodes subsequently inhibit the non-target words in the bilingual lexicon. Behaviorally, the absence of the main effect of prime congruency supports the model’s prediction as neither transliterated nor translated primes (both in different orthographies with reference to the target word) influenced the target word processing. However, this finding from the current study raises certain concerns on the phonological similarity between the prime and target words, as proposed in the BIA + model^10^. That is, according to the BIA + model, in addition to the orthographic overlap, the phonological and semantic similarities between the languages also contribute to bilingual visual word processing. Behavioral results from the current study show that, in the absence of orthographic similarity, the phonological overlap between the prime and target may not exert an influence on bilingual visual word processing. For instance, in the case of a transliterated (P + O–) prime-target dyad, both the prime and target shared the same phonology and semantics, differing only in their orthography. However, such prime-target dyads failed to reveal any response time (RT) advantage over the translation (P–O–) dyads, thus, raising concerns over the role of phonology in bilingual visual word processing, especially in the cross-script contexts. Similarly, from the ERP data, we expected an N250 effect arising from the difference in phonology between the two types of prime-target dyads used in this study. However, such an effect was not observed. Thus, based on both behavioral and electrophysiological data, we consider that in the absence of the orthographic overlap (such as in the current study), the phonological (and semantic) similarity between the primes and targets fail to show any processing advantage. The findings from this study, thus, support the BIA^8,9^ model, but not the BIA + model^10^ in cross-script visual word processing contexts.

## Method

### Participants

Eighteen Malayalam-English bilingual adults between 18 and 25 years of age (Mean = 21.4 years) were recruited to this study from the university community. Malayalam was their native language (L1) and English was their second language (L2). All participants started learning English before the age of 5 years with the commencement of kindergarten, and rated their proficiency in this language between 5 and 7 on a scale of 1–7, indicating ‘Good’ to ‘Native-like’ proficiency in speaking, listening, reading, and writing. None of them had any history or complaint of neurological, auditory, or language disorders. All of them had a normal or corrected-to-normal vision. The sample size is comparable with the studies that used similar methodology to investigate phonological processing using EEG^5,14,15,23,29,37^. The sample size was also adequate in detecting differences in ERPs between two conditions in a within-subject design with 80% power (using data simulation for cluster-based permutation tests^38^). The study was approved by the ethics committee at the College of Health Professions, Manipal Academy of Higher Education. Informed consent was obtained from all participants. All methods were performed in accordance with the relevant guidelines and regulations.

### Stimuli

A set of 100 English words (names of objects/items) were chosen for this study, of which 50 had unique translation equivalents in Malayalam (e.g., Table– /me:ʃa/), and the remaining did not have such translations in Malayalam (e.g., CAR-/car/). That is, the latter set of English words was borrowed to Malayalam such that they were written in Malayalam orthography, though spoken akin to English pronunciation (i.e., transliterated words: e.g., ‍-CAR). The English words were 3–11 letters in length and their transliterated and translated Malayalam counterparts were 2–5 letters in length. This difference in character length is expected as English is an alphabetic language, whereas, Malayalam is an alphasyllabary (more like a syllabic orthography). The translated and transliterated English words (to Malayalam) did not differ in their frequency (*t* = − 0.38, *p* = 0.7), word length (*t* = − 0.97, *p* = 0.33), bigram frequency (*t* = − 1.3, *p* = 0.19), as well as on the orthographic neighborhood density (*t* = − 1.01, *p* = 0.31) (English Lexicon Project). As the Malayalam words did not have information on these variables, we obtained the familiarity rating (on a scale of 5: 1—highly familiar, 2—familiar, 3—unfamiliar, 4—highly unfamiliar, & 5—never heard) of an initial corpus of Malayalam 170 words by five native speakers. From their ratings, we selected 50 words each that received either ‘highly familiar’ or ‘familiar’ rating from all the participants (who rated), to the translation and transliteration trials. These two sets of Malayalam words did not differ from each other in their familiarity rating (*t* = − 0.8, *p* = 0.42). The stimuli used in this study as well as their details are provided in the Supplementary material.

In this study, we specifically explored the influence of phonological similarity/dissimilarity of the primes on the target word processing. Therefore, we did not introduce any semantic controls (e.g., non-words). Thus, all the primes and targets were true words. We used four types of prime-stimulus relations such as: a) L2-L1 Phonologically congruent (i.e., English Loan Word_Prime_—Malayalam Transliteration Word_Target_: e.g.,car-{ka:r} ), b) L2-L1 Phonologically incongruent (i.e., English Unique Word_Prime_—Malayalam Translation Word_Target_: e.g., table— {me:ʃa}), c) L1-L2 Phonologically congruent (i.e., Malayalam Transliteration Word_Prime_—English Loan Word_Target_: e.g.,{ka:r}-CAR), and d) L1-L2 Phonologically incongruent (i.e., Malayalam Translation Word_Prime_—English Unique Word_Target_: e.g.,  {me:ʃa}—TABLE). Thus, it may be noted that both the prime and target always represented the same concept across all conditions, thus nullifying any effect of semantics. Further, the orthography of the prime and target was always different in all conditions. Only the phonology was different between the prime and target. In transliteration trials, the phonology of the prime and target matched each other, whereas, in the translation condition, it did not.

### Procedure

**Figure 3:**
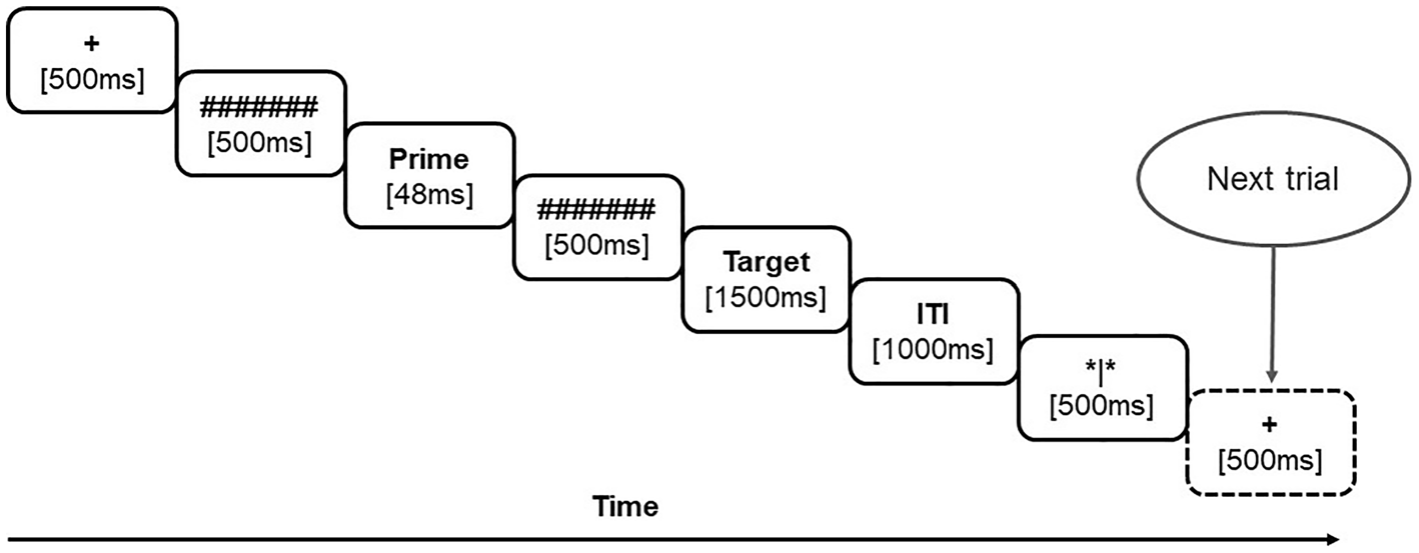
Scheme of an experimental trial.

The participants were seated ~ 100 cm away from the computer monitor (with a 60 Hz refresh rate) in a comfortable chair. Both the prime and target words were displayed in the center of a 24″ monitor as black letters on a white background in the Baraha font (Baraha 7.0). The presentation scheme of a trial is depicted in Fig. 3. Each trial began with the presentation of a fixation cross ( +) on the center of the screen for 500 ms which was then followed by a forward mask (######) for 500 ms. The prime was presented for 48 ms which was then replaced by a backward mask (######) for 500 ms. The target followed the backward mask and remained on the screen for 1500 ms or until the participant made a response. English words in the prime position were in lowercase (Block 1), and in the target position, were presented in upper case (Block 2). After the target word disappeared, an inter-trial interval in the form of a blank screen of 1000 ms was presented which in turn was followed by a ‘blink screen’ (*|*) for 500 ms where participants were allowed to blink, if desired. Their task was to press ‘1’ for the name words of natural objects (e.g., earth), and ‘2’ for man-made objects (e.g., computer) on the right-sided number keys of a standard computer keyboard. All participants performed the semantic judgment on the translated and transliterated prime-target pairs (N = 50 each) in both languages (total number of trials = 200). Half of the participants received Malayalam words as targets (with English primes) initially and the remaining received English targets first (with Malayalam primes). The experiment was designed and deployed with E-Prime experiment software (E-prime 2.0).

### EEG recording procedure

Continuous EEG was acquired with 30 Ag–AgCl scalp electrodes mounted in an electrode cap (Quick-Cap, Compumedics Neuroscan 4.5) in accordance with the standard 10/20 system. The ground electrode was positioned between FPz and Fz. Electrical activity from both the mastoids was recorded, with the left mastoid (M1) as the reference. Two electrodes positioned above and below the left eye measured the vertical eye movement (VEOG), and another electrode pair placed on the outer canthi of each eye measured the horizontal eye movements (HEOG). The impedance at each electrode was maintained below 5 kΩ. The signal from the scalp electrodes was amplified 20,000 times (SynAmps 2 amplifier, Compumedics), sampled at 1000 Hz, and low pass filtered at 100 Hz online.

### Data analysis

We used the EEGLAB^39^, ERPLAB^40^, and fieldtrip^41^ toolboxes running on MATLAB 2020a (Mathworks, Natik, MA, USA), for the data analysis. The continuous EEG data was (band-pass) filtered with windowed-sync finite impulse response (FIR) filter between 0.1 and 30 Hz with a roll-off of 12 dB/Octave. EEG was then divided into epochs of -100 to 800 ms duration relative to the target onset using ERPLAB. There were 4 conditions: (1) Phonology-congruent English_Prime_-Malayalam_Target_ (i.e., Malayalam transliterated words); (2) Phonology-incongruent English_Prime_-Malayalam_Target_ (i.e., Malayalam translated words); (3) Phonology-congruent Malayalam_Prime_-English_Target_ (i.e., English loan words); and (4) Phonology-incongruent Malayalam_Prime_-English_Target_ (i.e., English unique words). These epochs were baseline-corrected between -100 to 0 ms. The EEG data were then subjected to independent component analysis using EEGLAB. Subsequently, we used the automated classification tool (ICLabel^42^) to identify the noisy components in the EEG data. Components with > 80% threshold for ‘eye blinks’, ‘muscle’, and ‘channel noise’ were eliminated from each participant’s EEG data. Additionally, we removed all epochs across the participants that exceeded the amplitude of ± 80 µV at any time point (artifacts). Each participant had at least 80% artifact-free trials in each condition. Each participant’s artifact-free epochs belonging to each of these four conditions were averaged. Finally, we separately averaged the epochs of the four conditions across the participants to obtain the grand average of the four conditions.

### Statistical analyses of ERP data

The statistical analysis was done in two stages. The first analysis was literature-driven, whereby the electrodes and time ranges of interest were selected based on previous research^27,28,43,44^. The ERPs were averaged across 9 electrodes around Cz (CP4, C4, CP3, CPz, Cz, FC4, C3, FCz, FC3) and the amplitudes were calculated from three time-windows: 100–200 ms, 200–350 ms, and 350–550 ms. These amplitudes were subjected to a 2 × 2 repeated measures analysis of variance (ANOVA) with the factors Target language (English, Malayalam) and prime congruency (translated, transliterated).

The second analysis was completely data-driven as the literature-driven analysis in the previous step was limited only to the known ERP effects. To this end, non-parametric cluster-based permutation tests were used^45^. We computed a repeated measures 2 × 2 cluster-based permutation ANOVA with Target language (English, Malayalam) and prime congruency (translated, transliterated) as factors. The main effects and interactions were tested separately. The analysis included all the time points between 0 to 600 ms and all electrodes. First, a series of *t*-tests were computed at each electrode and time point. From these time points, where the waveforms differed significantly from each other was identified (*p* < 0.05, two-tailed) for each channel. Clusters were then formed by connecting the time points that showed a significant effect based on temporal and spatial adjacency. This was done separately for data points that showed positive and negative *t* values. Cluster level statistics were computed by adding together all *t* values within a cluster (mass *t* score). To control Type I errors due to multiple comparisons (30 channels × 600 time-points), a permutation approach was used. For this, a data-driven null hypothesis distribution was created by randomly swapping the stimuli labels within participants 5,000 times and computing mass *t* scores for each randomization. The mass *t* scores obtained in the first step were then compared with the null hypothesis distribution. The cluster was determined to be significant if it fell in the top 2.5 or bottom 2.5 percentile of the null hypothesis distribution.

## Supplementary Information

Supplementary Information.

## References

[1] Brysbaert M, Van Wijnendaele I (2003). The importance of phonological coding in visual word recognition: Further evidence from second-language processing. Psychol. Belg..

[2] Costa A, Santesteban M, Caño A (2005). On the facilitatory effects of cognate words in bilingual speech production. Brain Lang..

[3] Kroll JF, Bobb SC, Wodniecka Z (2006). Language selectivity is the exception, not the rule: Arguments against a fixed locus of language selection in bilingual speech. Bilingualism.

[4] Linck JA, Hoshino N, Kroll JF (2008). Cross-language lexical processes and inhibitory control. Ment. Lex..

[5] Zhou H, Chen B, Yang M, Dunlap S (2010). Language nonselective access to phonological representations: Evidence from Chinese-English bilinguals. Q. J. Exp. Psychol..

[6] Gollan TH, Forster KI, Frost R (1997). Translation priming with different scripts: Masked priming with cognates and noncognates in Hebrew-English bilinguals. J. Exp. Psychol. Learn Mem. Cogn..

[7] Hoshino N, Beatty-Martínez AL, Navarro-Torres CA, Kroll JF (2021). Do cross-language script differences enable bilinguals to function selectively when speaking in one language alone?. Front. Commun..

[8] Grainger, J. & Dijkstra, T. On the Representation and Use of Language Information in Bilinguals. in *Cognitive processing in bilinguals* (ed. Harris, R. J.) 207–220 (Elsevier, 1992).

[9] van Heuven WJB, Dijkstra T, Grainger J (1998). Orthographic neighborhood effects in bilingual word recognition. J. Mem. Lang..

[10] Dijkstra T, van Heuven WJB (2002). The architecture of the bilingual word recognition system: From identification to decision. Bilingualism.

[11] Dijkstra, T. & van Heuven, W. J. B. The BIA model and bilingual word recognition. in *Localist connectionist approaches to human cognition* (eds. Grainger, J. & Jacobs, A. M.) 189–225 (Lawrence Erlbaum Associates Publishers, 1998).

[12] Bijeljac-babic R, Biardeau A, Grainger J (1997). Masked orthographic priming in bilingual word recognition. Mem. Cogn..

[13] Duyck W (2005). Translation and associative priming with cross-lingual pseudohomophones: evidence for nonselective phonological activation in bilinguals. J. Exp. Psychol. Learn Mem. Cogn..

[14] Schoonbaert S, Holcomb PJ, Grainger J, Hartsuiker RJ (2011). Testing asymmetries in noncognate translation priming: Evidence from RTs and ERPs. Psychophysiology.

[15] Peleg O, Degani T, Raziq M, Taha N (2020). Cross-lingual phonological effects in different-script bilingual visual-word recognition. Second Lang. Res..

[16] Forster KI, Davis C (1984). Repetition priming and frequency attenuation in lexical access. J. Exp. Psychol. Learn Mem. Cogn..

[17] Forster, K. I., Mohan, K. & Hector, J. The mechanics of masked priming. in *Masked Priming: The State of the Art* (eds. Kinoshita, S. & Lupker, S. J.) 2–20 (Taylor & Francis, 2003).

[18] Van Wijnendaele I, Brysbaert M (2002). Visual word recognition in bilinguals: Phonological priming from the second to the first language. J. Exp. Psychol. Hum. Percept. Perform..

[19] Basnight-Brown DM, Altarriba J (2007). Differences in semantic and translation priming across languages: The role of language direction and language dominance. Mem. Cognit..

[20] Voga M, Grainger J (2007). Cognate status and cross-script translation priming. Mem. Cognit..

[21] Grainger J, Ferrand L (1996). Masked orthographic and phonological priming in visual word recognition and naming: Cross-task comparisons. J. Mem. Lang..

[22] Bentin S, Mouchetant-Rostaing Y, Giard MH, Echallier JF, Pernier J (1999). ERP manifestations of processing printed words at different psycholinguistic levels: Time course and scalp distribution. J. Cogn. Neurosci..

[23] Chauncey K, Holcomb PJ, Grainger J (2008). Effects of stimulus font and size on masked repetition priming: An event-related potentials (ERP) investigation. Lang. Cogn. Process..

[24] Dufau S, Grainger J, Holcomb PJ (2008). An ERP investigation of location invariance in masked repetition priming. Cogn. Affect. Behav. Neurosci..

[25] Grainger J, Holcomb PJ (2009). Watching the word go by: On the time-course of component processes in visual word recognition. Lang. Linguist. Compass.

[26] Grainger J, Kiyonaga K, Holcomb PJ (2006). The time course of orthographic and phonological code activation. Psychol. Sci..

[27] Emmorey K, Holcomb PJ, Midgley KJ (2021). Masked ERP repetition priming in deaf and hearing readers. Brain Lang..

[28] Holcomb PJ, Grainger J (2007). Exploring the temporal dynamics of visual word recognition in the masked repetition priming paradigm using event-related potentials. Brain Res..

[29] Hoshino N, Midgley KJ, Holcomb PJ, Grainger J (2010). An ERP investigation of masked cross-script translation priming. Brain Res..

[30] Midgley KJ, Holcomb PJ, Grainger J (2009). Language effects in second language learners and proficient bilinguals investigated with event-related potentials. J. Neurolinguistics.

[31] Chauncey K, Grainger J, Holcomb P (2008). Code-switching effects in bilingual word recognition: A masked priming study with event-related potentials. Brain Lang..

[32] Hoversten LJ, Brothers T, Swaab TY, Traxler MJ (2017). Early processing of orthographic language membership information in bilingual visual word recognition: Evidence from ERPs. Neuropsychologia.

[33] Chang L-Y, Chen Y-C, Perfetti CA (2018). GraphCom: A multidimensional measure of graphic complexity applied to 131 written languages. Behav. Res..

[34] Brysbaert M, Van Dyck G, Van de Poel M (1999). Visual word recognition in bilinguals: Evidence from masked phonological priming. J. Exp. Psychol. Hum. Percept. Perform..

[35] Jouravlev O, Lupker SJ, Jared D (2014). Cross-language phonological activation: Evidence from masked onset priming and ERPs. Brain Lang..

[36] Carreiras M, Perea M, Vergara M, Pollatsek A (2009). The time course of orthography and phonology: ERP correlates of masked priming effects in Spanish. Psychophysiology.

[37] Hauk O, Davis MH, Ford M, Pulvermüller F, Marslen-Wilson WD (2006). The time course of visual word recognition as revealed by linear regression analysis of ERP data. Neuroimage.

[38] Wang C, Zhang Q (2021). Word frequency effect in written production: Evidence from ERPs and neural oscillations. Psychophysiology.

[39] Delorme A, Makeig S (2004). EEGLAB: an open source toolbox for analysis of single-trial EEG dynamics including independent component analysis. J. Neurosci. Methods.

[40] Lopez-Calderon, J. & Luck, S. J. ERPLAB: an open-source toolbox for the analysis of event-related potentials. *Front. Hum. Neurosci.* **8**, (2014). 10.3389/fnhum.2014.00213 PMC3995046 24782741

[41] Oostenveld R, Fries P, Maris E, Schoffelen J-M (2011). FieldTrip: Open source software for advanced analysis of MEG, EEG, and invasive electrophysiological data. Comput. Intell. Neurosci..

[42] Pion-Tonachini L, Kreutz-Delgado K, Makeig S (2019). ICLabel: An automated electroencephalographic independent component classifier, dataset, and website. Neuroimage.

[43] Wu Y, Duan R, Zhao S, Tsang Y-K (2020). Processing ambiguous morphemes in Chinese compound word recognition: Behavioral and ERP evidence. Neuroscience.

[44] Morris J, Stockall L (2012). Early, equivalent ERP masked priming effects for regular and irregular morphology. Brain Lang..

[45] Maris E, Oostenveld R (2007). Nonparametric statistical testing of EEG- and MEG-data. J. Neurosci. Methods.

