# Supplementary material for: A prime-masked ERP investigation on phonology in visual word processing among bilingual speakers of alphasyllabic and alphabetic orthographies
Source: Sci Rep. 2022 Jun 14;12:9870. doi: 10.1038/s41598-022-13654-8 (PMC9198037; doi:10.1038/s41598-022-13654-8)
Supplement: Supplementary file 1 — Supplementary Information. [file 41598_2022_13654_MOESM1_ESM.docx]

**Stimuli**

**English words loaned to Malayalam**

| **English loaned word** | **Length** | **Frequency** | **Orthographic neighborhood density** | **Phonological neighborhood density** | **Mean bigram frequency** | **Phoneme count** | **Syllable count** | **Malayalam script** |
| --- | --- | --- | --- | --- | --- | --- | --- | --- |
| Apple | 5 | 11.09 | 2 | 4 | 1,393 | 3 | 2 | ആപ്പിൾ |
| Bag | 3 | 9.7 | 20 | 27 | 780.5 | 3 | 1 | ബാഗ് |
| Battery | 7 | 10.22 | 2 | 0 | 2,803.50 | 6 | 3 | ബാറ്ററി |
| Bike | 4 | 10.02 | 9 | 22 | 534.667 | 3 | 1 | ബൈക്ക് |
| Bill | 4 | 11.66 | 16 | 34 | 1,221.67 | 3 | 1 | ബിൽ |
| Biscuit | 7 | 6.39 | 0 | 1 | 1,253.67 | 6 | 2 | ബിസ്കറ്റ് |
| Blackboard | 10 | 6.13 | 0 | 0 | 1,157.11 | 8 | 2 | ബ്ലാക്ക്ബോർഡ് |
| Blade | 5 | 9.16 | 5 | 9 | 1,712.75 | 4 | 1 | ബ്ലേഡ് |
| Brush | 5 | 8.64 | 3 | 4 | 1,000.50 | 4 | 1 | ബ്രഷ് |
| Bucket | 6 | 8.11 | 2 | 2 | 831.2 | 5 | 2 | ബക്കറ്റ് |
| Button | 6 | 10.32 | 1 | 5 | 1,620.80 | 4 | 2 | ബട്ടൺ |
| Cake | 4 | 8.74 | 18 | 24 | 1,010.67 | 3 | 1 | കേക്ക് |
| Calendar | 8 | 9.14 | 0 | 1 | 2,480 | 7 | 3 | കലണ്ടർ |
| Camera | 6 | 9.82 | 0 | 0 | 2,752.20 | 5 | 2 | ക്യാമറ |
| Car | 3 | 11.36 | 15 | 21 | 2,384 | 3 | 1 | കാർ |
| Carrot | 6 | 7.26 | 1 | 6 | 1,678.80 | 5 | 2 | കാരറ്റ് |
| Chalk | 5 | 7.41 | 0 | 13 | 1,458 | 3 | 1 | ചോക്ക് |
| Charger | 7 | 7.7 | 4 | 4 | 2,217.67 | 5 | 2 | ചാർജർ |
| Clip | 4 | 8.89 | 6 | 10 | 1,284.33 | 4 | 1 | ക്ലിപ്പ് |
| Computer | 8 | 12.44 | 3 | 0 | 2,351.86 | 8 | 3 | കമ്പ്യുട്ടർ |
| Fan | 3 | 10.53 | 14 | 26 | 1,955.50 | 3 | 1 | ഫാൻ |
| Fridge | 6 | 7.37 | 2 | 2 | 1,089.40 | 4 | 1 | ഫ്രിഡ്ജ് |
| Hanger | 6 | 6.86 | 6 | 5 | 3,184 | 4 | 2 | ഹാങ്ങർ |
| Helmet | 6 | 8.65 | 0 | 0 | 1,336.60 | 6 | 2 | ഹെൽമെറ്റ് |
| Lens | 4 | 9.14 | 9 | 18 | 3,029.33 | 4 | 1 | ലെന്‍സ് |
| Orange | 6 | 9.5 | 1 | 2 | 2,895.40 | 5 | 2 | ഓറഞ്ച് |
| Pants | 5 | 6.28 | 11 | 16 | 2,533 | 4 | 1 | പാന്റ്സ് |
| Pencil | 6 | 8.01 | 0 | 1 | 1,859 | 5 | 2 | പെൻസിൽ |
| Photo | 5 | 9.54 | 0 | 2 | 1,004.25 | 4 | 2 | ഫോട്ടോ |
| Plastic | 7 | 10.18 | 1 | 1 | 2,480.33 | 7 | 2 | പ്ലാസ്റ്റിക് |
| Plug | 4 | 9.66 | 3 | 7 | 558.33 | 4 | 1 | പ്ളഗ് |
| Purse | 5 | 7.52 | 6 | 19 | 1,312.50 | 3 | 1 | പേഴ്സ് |
| Radio | 5 | 11.18 | 2 | 2 | 2,136.50 | 5 | 3 | റേഡിയോ |
| Remote | 6 | 10.21 | 2 | 1 | 2,363.40 | 5 | 2 | റിമോട്ട് |
| Ribbon | 6 | 8.18 | 1 | 3 | 1,762.60 | 5 | 2 | റിബൺ |
| Road | 4 | 11.1 | 6 | 36 | 1,204.67 | 3 | 1 | റോഡ് |
| Screw | 5 | 9.16 | 2 | 3 | 1,631 | 4 | 1 | സ്ക്രൂ |
| Shoes | 5 | 9.36 | 4 | 21 | 1,781 | 3 | 1 | ഷൂസ് |
| Soap | 4 | 8.99 | 6 | 21 | 691.66 | 3 | 1 | സോപ്പ് |
| Soup | 4 | 8.7 | 4 | 17 | 954 | 3 | 1 | സൂപ്പ് |
| Speaker | 7 | 9.7 | 1 | 2 | 1,949.67 | 5 | 2 | സ്പീക്കര്‍ |
| Switch | 6 | 10.57 | 4 | 11 | 919.4 | 4 | 1 | സ്വിച്ച് |
| Tap | 3 | 9.34 | 19 | 21 | 1,375 | 3 | 1 | ടാപ്പ് |
| Tar | 3 | 9.02 | 16 | 20 | 2,421 | 3 | 1 | ടാർ |
| Telephone | 9 | 10.58 | 0 | 0 | 2,355.75 | 7 | 3 | ടെലിഫോൺ |
| Toothpaste | 10 | 6.57 | 0 | 0 | 1,671.33 | 7 | 2 | ടൂത്ത്പേസ്റ്റ് |
| Truck | 5 | 9.23 | 4 | 6 | 934.5 | 4 | 1 | ട്രക്ക് |
| Uniform | 7 | 9 | 0 | 0 | 1,300.33 | 8 | 3 | യൂണിഫോം |
| Video | 5 | 11.61 | 0 | 0 | 1,116.50 | 5 | 3 | വീഡിയോ |
| Wire | 4 | 9.71 | 16 | 22 | 1,939.67 | 3 | 1 | വയർ |

**Unique English words**

| **English unique words** | **Length** | **Frequency** | **Orthographic neighborhood density** | **Phonological neighborhood density** | **Mean bigram frequency** | **Phoneme count** | **Syllable count** | **Malayalam translation** | **Malayalam script** |
| --- | --- | --- | --- | --- | --- | --- | --- | --- | --- |
| Air | 3 | 11.3 | 5 | 12 | 864 | 2 | 1 | vaːju | വായു |
| Ant | 3 | 7.92 | 7 | 9 | 3,250.50 | 3 | 1 | uRumb ̂ | ഉറുമ്പ് |
| Ball | 4 | 10.55 | 19 | 30 | 1,819.33 | 3 | 1 | pant̪ ̂ | പന്ത് |
| Banana | 6 | 7.96 | 0 | 0 | 2,119.40 | 6 | 3 | pazham | പഴം |
| Bangle | 6 | 3.71 | 5 | 6 | 2,406.20 | 5 | 2 | val̥a | വള |
| Book | 4 | 12.16 | 13 | 20 | 639.67 | 3 | 1 | pustəkʌm | പുസ്തകം |
| Bottle | 6 | 9.83 | 2 | 10 | 1,235.40 | 4 | 2 | kuppɪ | കുപ്പി |
| Butterfly | 9 | 7.49 | 0 | 0 | 1,855 | 7 | 3 | tʃɪt̪raʃalabʰam | പൂമ്പാറ്റ |
| Chair | 5 | 9.83 | 3 | 27 | 1,097.50 | 3 | 1 | kaseːra | കസേര |
| Clock | 5 | 10.1 | 8 | 13 | 909.75 | 4 | 1 | gʰadikaːram | ഘടികാരം |
| Cloud | 5 | 8.34 | 1 | 5 | 899 | 5 | 1 | meːgʰam | മേഘം |
| Crab | 4 | 6.88 | 7 | 9 | 1,637.33 | 4 | 1 | ɲand ̂ | ഞണ്ട് |
| Curtain | 7 | 7.57 | 2 | 3 | 2,117.50 | 4 | 2 | t̪iraʃiːla | തിരശ്ശീല |
| Door | 4 | 10.89 | 6 | 35 | 1,422.67 | 3 | 1 | vɑːtɪl | വാതിൽ |
| Dress | 5 | 9.42 | 3 | 4 | 2,749.50 | 4 | 1 | vast̪ram | വസ്ത്രം |
| Ear | 3 | 9.33 | 11 | 19 | 2,390 | 2 | 1 | tʃevɪ | ചെവി |
| Earring | 7 | 6.03 | 5 | 0 | 3,174.33 | 5 | 2 | kammal | കമ്മൽ |
| Eye | 3 | 10.62 | 8 | 7 | 209 | 1 | 1 | kan̥n̥ ̂ | കണ്ണ് |
| Hair | 4 | 10.66 | 5 | 32 | 957.67 | 3 | 1 | mudɪ | മുടി |
| Hand | 4 | 11.73 | 9 | 13 | 2,130 | 4 | 1 | kəɪ | കൈ |
| Hat | 3 | 9.37 | 20 | 33 | 2,663 | 3 | 1 | tɔːpɪ | തൊപ്പി |
| Head | 4 | 11.6 | 13 | 28 | 1,538.33 | 3 | 1 | t̪ala | തല |
| House | 5 | 11.55 | 5 | 11 | 1,592 | 3 | 1 | vɪːd ̂ | വീട് |
| Ladder | 6 | 7.99 | 4 | 23 | 2,407.80 | 4 | 2 | e:Ni | ഏണി |
| Leg | 3 | 9.78 | 12 | 13 | 1,925 | 3 | 1 | kaːl | കാല് |
| Light | 5 | 11.48 | 7 | 34 | 1,094.25 | 3 | 1 | prakaːʃam | പ്രകാശം |
| Lock | 4 | 9.81 | 15 | 29 | 1,020 | 3 | 1 | puːt | പൂട്ട് |
| Needle | 3 | 10.11 | 16 | 33 | 3,858 | 3 | 1 | sutʃɪ | സൂചി |
| Nose | 4 | 9.54 | 12 | 25 | 1,225 | 3 | 1 | muːkk ̂ | മൂക്ക് |
| Onion | 5 | 7.85 | 2 | 0 | 3,309.50 | 5 | 2 | ul̥l̥ɪ | ഉള്ളി |
| Paper | 5 | 11.06 | 4 | 10 | 2,449 | 4 | 2 | kadalaːs ̂ | കടലാസ് |
| Parrot | 6 | 7.47 | 1 | 9 | 1,537.80 | 5 | 2 | t̪at̪t̪a | തത്ത |
| Pomegranate | 11 | 4.51 | 0 | 0 | 2,181.10 | 10 | 4 | maːt̪ʌl̥anaːraŋa | മാതളനാരങ്ങ |
| Potato | 6 | 8.06 | 0 | 0 | 1,880.80 | 6 | 3 | urulakkil̪aŋ | കിഴങ്ങ് |
| Ring | 4 | 10.69 | 10 | 23 | 4,562.67 | 3 | 1 | moːt̪ɪram | മോതിരം |
| Spectacles | 10 | 5.61 | 0 | 0 | 1,862 | 9 | 3 | kannada | കണ്ണട |
| Star | 4 | 11.31 | 10 | 8 | 2,802.33 | 4 | 1 | n̥aksat̪Ram | നക്ഷത്രം |
| Sweet | 5 | 9.64 | 7 | 7 | 726 | 4 | 1 | mʌðurʌm | മധുരം |
| Swing | 5 | 8.95 | 6 | 16 | 2,869.50 | 4 | 1 | uːɲaːl | ഊഞ്ഞാൽ |
| Sword | 5 | 9.72 | 2 | 18 | 979 | 4 | 1 | vaːǃ | വാൾ |
| Table | 5 | 10.93 | 4 | 6 | 1,869.75 | 4 | 2 | meːʃa | മേശ |
| Tail | 3 | 10.16 | 18 | 25 | 1,276.50 | 3 | 1 | va:l | വാല്‍ |
| Tomato | 6 | 7.66 | 0 | 0 | 1,962 | 6 | 3 | t̪akkaːlɪ | തക്കാളി |
| Tongue | 6 | 9.61 | 0 | 15 | 2,134.20 | 3 | 1 | n̥aːvu | നാവ് |
| Tree | 4 | 10.21 | 4 | 9 | 2,366.67 | 3 | 1 | vRɪks̥am | വൃക്ഷം |
| Umbrella | 8 | 7.56 | 0 | 0 | 1,649.29 | 7 | 3 | kuda | കുട |
| Well | 4 | 13.22 | 12 | 24 | 1,278.33 | 3 | 1 | kin̥aR | കിണർ |
| Window | 6 | 10.91 | 0 | 0 | 2,079.80 | 5 | 2 | dʒɑnal | ജനല്‍ |
| Woman | 5 | 11.1 | 2 | 2 | 1,679.50 | 5 | 2 | st̪rɪ | സ്ത്രീ |

**English words borrowed to Malayalam**

| English word | Malayalam transliteration | Mean familiarity rating | Syllable count in Malayalam |
| --- | --- | --- | --- |
| Apple | ആപ്പിൾ | 1 | 2 |
| Bag | ബാഗ് | 1.6 | 1 |
| Battery | ബാറ്ററി | 1 | 3 |
| Bike | ബൈക്ക് | 1.8 | 1 |
| Bill | ബിൽ | 1.6 | 1 |
| Biscuit | ബിസ്കറ്റ് | 1 | 2 |
| Blackboard | ബ്ലാക്ക്ബോർഡ് | 1.8 | 2 |
| Blade | ബ്ലേഡ് | 1.4 | 1 |
| Brush | ബ്രഷ് | 1.2 | 1 |
| Bucket | ബക്കറ്റ് | 1 | 2 |
| Button | ബട്ടൺ | 1.2 | 2 |
| Cake | കേക്ക് | 1 | 1 |
| Calendar | കലണ്ടർ | 1.8 | 3 |
| Camera | ക്യാമറ | 1.4 | 3 |
| Car | കാർ | 1.6 | 1 |
| Carrot | കാരറ്റ് | 1 | 2 |
| Chalk | ചോക്ക് | 1.2 | 1 |
| Charger | ചാർജർ | 1.6 | 2 |
| Clip | ക്ലിപ്പ് | 1.2 | 1 |
| Computer | കമ്പ്യുട്ടർ | 1 | 3 |
| Fan | ഫാൻ | 1 | 1 |
| Fridge | ഫ്രിഡ്ജ് | 1.2 | 1 |
| Hanger | ഹാങ്ങർ | 1.6 | 2 |
| Helmet | ഹെൽമെറ്റ് | 1 | 2 |
| Lens | ലെന്സ് | 1.8 | 1 |
| Orange | ഓറഞ്ച് | 1 | 2 |
| Pants | പാന്റ്സ് | 1.4 | 1 |
| Pencil | പെൻസിൽ | 1.6 | 2 |
| Photo | ഫോട്ടോ | 1 | 2 |
| Plastic | പ്ലാസ്റ്റിക് | 1 | 2 |
| Plug | പ്ളഗ് | 1 | 1 |
| Purse | പേഴ്സ് | 1.2 | 1 |
| Radio | റേഡിയോ | 1.2 | 3 |
| Remote | റിമോട്ട് | 1.4 | 2 |
| Ribbon | റിബൺ | 1.2 | 2 |
| Road | റോഡ് | 1 | 1 |
| Screw | സ്ക്രൂ | 1.2 | 1 |
| Shoes | ഷൂസ് | 1.2 | 1 |
| Shoes | ഷൂസ് | 1.4 | 1 |
| Soap | സോപ്പ് | 1.8 | 1 |
| Soup | സൂപ്പ് | 1.2 | 1 |
| Switch | സ്വിച്ച് | 1.2 | 1 |
| Tap | ടാപ്പ് | 1.6 | 1 |
| Tar | ടാർ | 1 | 1 |
| Telephone | ടെലിഫോൺ | 1.4 | 3 |
| Toothpaste | ടൂത്ത്പേസ്റ്റ് | 1 | 2 |
| Truck | ട്രക്ക് | 1 | 1 |
| Uniform | യൂണിഫോം | 1.2 | 3 |
| Video | വീഡിയോ | 1.6 | 3 |
| Wire | വയർ | 1.2 | 2 |

**Unique Malayalam words**

| Malayalam unique word | Transcription in Malayalam | English translation | Mean familiarity rating | Syllable count in Malayalam |
| --- | --- | --- | --- | --- |
| വായു | vaːju | Air | 2 | 2 |
| ഉറുമ്പ് | uRumb ̂ | Ant | 1 | 2 |
| പന്ത് | pant̪ ̂ | Ball | 1.6 | 1 |
| വള | val̥a | Bangle | 1.2 | 2 |
| പുസ്തകം | pustəkʌm | Book | 1.4 | 3 |
| കുപ്പി | kuppɪ | Bottle | 1 | 2 |
| പൂമ്പാറ്റ | pu:mpa:tta | Butterfly | 2 | 3 |
| മാല | maːla | Chain | 1 | 2 |
| കസേര | kaseːra | Chair | 1.2 | 3 |
| ഘടികാരം | gʰadikaːram | Clock | 2 | 4 |
| വസ്ത്രം | vast̪ram | Cloth | 1.6 | 1 |
| മേഘം | meːgʰam | Cloud | 1.8 | 2 |
| ഞണ്ട് | ɲand ̂ | Crab | 1 | 1 |
| തിരശ്ശീല | t̪iraʃiːla | Curtain | 2 | 4 |
| വാതിൽ | vɑːtɪl | Door | 1 | 2 |
| ചെവി | tʃevɪ | Ear | 1 | 2 |
| കമ്മൽ | kammal | Ear-ring | 1 | 2 |
| കണ്ണ് | kan̥n̥ ̂ | Eye | 1 | 1 |
| പഴം | pazham | Fruit | 1 | 2 |
| മുടി | mudɪ | Hair | 1 | 2 |
| കൈ | kəɪ | Hand | 1 | 1 |
| തൊപ്പി | tɔːpɪ | Hat | 1.2 | 2 |
| തല | t̪ala | Head | 1 | 2 |
| വീട് | vɪːd ̂ | House | 1 | 1 |
| ഏണി | e:Ni | Ladder | 2 | 2 |
| കാല് | kaːl | Leg | 1 | 1 |
| പ്രകാശം | prakaːʃam | Light | 1.6 | 3 |
| പൂട്ട് | puːt | Lock | 1.6 | 1 |
| വാല്‍ | va:l | Tail | 1.6 | 1 |
| കണ്ണാടി | kannadɪ | Mirror | 1 | 3 |
| സൂചി | sutʃɪ | Needle | 1 | 2 |
| മൂക്ക് | muːkk ̂ | Nose | 1 | 1 |
| ഉള്ളി | ul̥l̥ɪ | Onion | 1 | 2 |
| കടലാസ് | kadalaːs ̂ | Paper | 1.6 | 3 |
| തത്ത | t̪at̪t̪a | Parrot | 1.2 | 2 |
| മാതളനാരങ്ങ | maːt̪ʌl̥anaːraŋa | Pomegranate | 1.8 | 6 |
| കിഴങ്ങ് | Urulakkizaŋ | Potato | 1 | 2 |
| മോതിരം | moːt̪ɪram | Ring | 1 | 3 |
| നക്ഷത്രം | n̥aksat̪Ram | Star | 1.4 | 3 |
| മധുരം | mʌðurʌm | Sweet | 1.2 | 3 |
| ഊഞ്ഞാൽ | uːɲaːl | Swing | 1 | 2 |
| വാൾ | vaːǃ | Sword | 1.8 | 1 |
| മേശ | meːʃa | Table | 1.2 | 2 |
| തക്കാളി | t̪akkaːlɪ | Tomato | 1 | 3 |
| നാവ് | n̥aːvu | Tongue | 1.4 | 1 |
| വൃക്ഷം | vRɪks̥am | Tree | 1.6 | 2 |
| കുട | kuda | Umbrella | 1 | 2 |
| കിണർ | kin̥aR | Well | 1.4 | 2 |
| ജനല്‍ | dʒɑnal | Window | 2 | 2 |
| സ്ത്രീ | st̪ri: | Woman | 1.4 | 1 |
